# Light-Dependent Changes in the Spatial Localization of Metabolites in *Solenostemon scutellarioides* (Coleus Henna) Visualized by Matrix-Free Atmospheric Pressure Electrospray Laser Desorption Ionization Mass Spectrometry Imaging

**DOI:** 10.3389/fpls.2018.01348

**Published:** 2018-09-19

**Authors:** Patrick A. McVey, Liza E. Alexander, Xinyu Fu, Bo Xie, Katherine-Jo Galayda, Basil J. Nikolau, Robert S. Houk

**Affiliations:** ^1^Department of Chemistry, Iowa State University, Ames, IA, United States; ^2^Ames Laboratory-US DOE, Ames, IA, United States; ^3^Center for Metabolic Biology, Iowa State University, Ames, IA, United States; ^4^Roy J. Carver Department of Biochemistry, Biophysics and Molecular Biology, Iowa State University, Ames, IA, United States

**Keywords:** matrix-free imaging, mass spectrometry imaging, electrospray laser desorption ionization, shading, Coleus (*Solenostemon scuttellarioides*), anthocyanin, flavonoid, source-to-sink

## Abstract

The visualization of foliage color in plants provides immediate insight into some of the compounds that exist in the leaf. However, many non-colored compounds are also present; their cellular distributions are not readily identifiable optically. In this study we evaluate the applicability of mass spectrometry imaging (MSI) via electrospray laser desorption ionization (ELDI) to reveal the spatial distribution of metabolites. ELDI-MSI is a matrix free, atmospheric pressure ionization method that utilizes a UV laser coupled with supplemental ionization by electrospray. We specifically applied ELDI-MSI to determine the spatial distribution of metabolites in Coleus Henna half leaves that were grown with half-sections either fully illuminated or shaded. We monitored dynamic changes in the spatial distribution of metabolites in response to the change of illumination every 7 days for a 28 day period. A novel source-sink relationship was observed between the 2 halves of the experimental leaf. Furthermore, Coleus Henna leaves present visually recognizable sectors associated with the differential accumulation of flavonoids. Thus, we correlated the effect of differential illumination and presence or absence of flavonoids with metabolic changes revealed by the accumulation of carbohydrates, amino acids, and organic acids. The results show the potential of ELDI-MSI to provide spatial information for a variety of plant metabolites with little sample preparation.

## Introduction

Traditional mass spectrometry (MS) based methods of metabolite analysis require extraction of metabolites from biological samples, followed by chromatography. Spatial distribution data on metabolites are lost in these procedures. Recently, mass spectrometry imaging (MSI) has made a significant impact in filling this gap and has contributed to more refined understanding of many areas of biology (Amstalden van Hove et al., 2011; Lee et al., 2012; Angel and Caprioli, 2013; Nilsson et al., 2015; Shroff et al., 2015). Interrogating the location of metabolites in multicellular organisms provides insight into the “sharing” of metabolic processes among different cell types.

Several ionization sources for MSI have been introduced. Each has its own unique advantages and limitations. Matrix assisted laser desorption ionization (MALDI) is the most widely used MSI technique in biology and can obtain spatial resolution as high as ~1 μm (Zavalin et al., 2015; Kompauer et al., 2017). MALDI performed in vacuum, coupled with different applied matrices, allows for the detection of a variety of metabolites (Klein et al., 2015; Shroff et al., 2015). However, the application of the matrix can complicate the use of MALDI especially for metabolites with low *m/z* values (<1000 Da) (Boughton et al., 2016), such as organic acids, sugars and amino acids. Laser ablation electrospray ionization (LAESI) generally uses an infrared laser to ablate particles from a biological surface, with water in the sample being the main IR absorber. The ablated plant particles undergo post-ionization via interaction with the ESI droplets before entering the mass spectrometer. As generally practiced, LAESI allows ~200 μm lateral resolution, while requiring little sample preparation because there is no applied matrix (Nemes and Vertes, 2007). Similar to the aforementioned methods, matrix assisted laser desorption electrospray ionization (MALDESI) utilizes an applied matrix to absorb radiation from an IR laser coupled with post-ionization via ESI; water ice is an effective matrix for MALDESI (Robichaud et al., 2014).

In the present study, electrospray laser desorption ionization (ELDI) with an ultraviolet laser (wavelength 355 nm) coupled with electrospray (Shiea et al., 2005), was used to analyze the spatial distribution of metabolites in plant samples. In this study, we applied ELDI to Coleus (*Solenostemon scuttellarioides*) leaves that were grown with only half of the leaf surface illuminated. Coleus is a member of the *Labiatae* (mint) family, and different varieties are generally grown as ornamentals. The Coleus variety Henna is known for its serrated foliage. Its upper side is a unique uniform chartreuse to copper color. The underside is dark burgundy. These colors are primarily due to anthocyanin pigments that accumulate in these plants. The color intensity can be affected by illumination, temperature, and other environmental conditions (Lebowitz, 2011).

The present work evaluates ELDI-MSI to study the distribution of anthocyanins, flavonoids, and small metabolites (<1000 Da), including carbohydrates, organic acids and amino acids in Coleus leaves. The effects of different states of illumination during growth are assessed.

## Materials and methods

### Plant growth, maintenance, and light conditions

*Solenostemon scuttellariodes* (Coleus Henna spp.) adult plants were obtained from Stam Greenhouse (Oskaloosa, IA). All plants were transferred to LC1 Sunshine Mix soil (Sun Gro Horticulture, Bellevue, WA), watered weekly, and grown in a growth room at 22°C under continuous illumination (2,568 Lux or photosynthetic photon flux density 100 μmol of photons m^–2^ sec^–1^).

For the light regulated experiments, half (through the leaf vein) of each leaf was covered length-wise with aluminum foil (shiny side up). The edges were sealed with scotch tape to ensure the covered region had limited light exposure. The leaves were harvested from the same plant at 1-week intervals for 4 weeks. In all imaging and profiling experiments, three biological replicates (each replicate being images of three plants) were used for MS imaging, optical imaging and metabolic profiling of all metabolites.

Leaves for anthocyanin and non-targeted metabolite profile analysis were harvested each week, cut through the middle vein to separate the light-exposed surface from the shaded/dark-surface, and immediately flash-frozen using liquid nitrogen. The samples were then dried using a vacuum lyophilizer and pulverized using a Mixer Mill 301 (Retsch GmbH, Germany) in 2 mL Eppendorf tubes prior to extraction.

For MS imaging, samples were gently cut from both the foil-covered and uncovered edge of the leaf. Sections measuring ~12 mm by 8 mm were immediately analyzed using ELDI. Leaf edges were also visualized under a light microscope.

### Tissue sectioning and microscopy

For microscopy only, leaf edges were hand-sectioned using a vibratome (TPI-3000; www.tedpella.com) at 70–100 μm. The sections were mounted in water and visualized under bright-field using a BH40 compound microscope (Olympus, www.olympus-global.com) equipped with Axio Vision software (Carl Zeiss Inc., Thorwood, NY). Additionally, a fresh leaf was ablated and the depth of the ablation trenches were measured using a Keyence VHX-5000 Digital Microscope.

### Anthocyanin extraction

Compounds were extracted from 5 mg of lyophilized Henna leaf tissue using 300 μL of methanol/water/acetic acid (85:15:0.5; v/v/v) and sonicated for 1 h. Samples were then incubated in dark at 4°C for 2 h (Wu et al., 2004). The samples were centrifuged for 5 min at 13,000 rpm and the liquid phase was filtered twice using 13 mm × 0.45 μm Teflon Syringe filters (Supelco, PA) for HPLC MS analysis.

### HPLC-MS and MS/MS analysis of anthocyanins

The chromatographic separations were performed with an Agilent Technologies 1100 series HPLC. This was coupled with an Agilent Technologies Mass Selective Trap SL detector, equipped with an electrospray ionization (ESI) source with an autosampler/injector and diode array detector (DAD) for LC-MS analysis. A silica-based reverse-phase C18 Atlantis T3 column (2.1 × 150 mm, 3 μm, Waters, Milford, MA) was used for separation. Elution was performed using mobile phases containing 5% formic acid in LC-MS grade water or methanol (MeOH). The flow rate was kept at 0.2 mL/min and the DAD detection was at UV Vis wavelength 520 nm. After 5 μL of sample injection, a gradient was used as follows: 0–6 min, 0–20% MeOH; 6–40 min, 20–50% MeOH; 40–44 min, 50–50% MeOH; 44–48 min, 50–00% MeOH 48–52 min, 100% MeOH; 52–56 min, 100–0% MeOH. The column effluent was then introduced via ESI into an Agilent 6210 MSD time-of- flight mass spectrometer operating in positive mode. The ESI capillary voltage was +3.0 kV, nitrogen gas temperature was set to 350°C, drying gas flow rate was 11 L/min, nebulizer gas pressure was 35 psi, skimmer was 65 V, and OCT RF was 250 V. Mass spectra from *m/*z 100 to 2000 were collected and analyzed using Agilent ChemStation Data Analysis. Individual anthocyanin peak areas were generated by Quant Analysis and used to compare different levels of anthocyanins quantitatively. The structure for each anthocyanin was identified from detailed MS/MS analysis performed at various collision energies.

### Non-targeted metabolite profile analysis by GC-MS

Metabolite extracts were prepared as described previously (Schmidt et al., 2011). Extracts were prepared from 2 mg of lyophilized leaf tissue. The extracts were spiked with two internal standards: 10 μg of ribitol and 5 μg nonadecanoic acid for polar and non-polar fractions, respectively. To 5 mg of lyophilized tissue, 0.35 ml of hot methanol (60°C) was added. The sample was incubated at the same temperature for 10 min, followed by sonication for 10 min at full power. To this slurry, 0.35 ml of chloroform and 0.3 ml of water were added and the mixture was vortexed for 1–3 min. After centrifugation for 5 min at 13,000 g, 200 μl of the upper phase (polar fraction) and 200 μl of the lower phase (non-polar fraction) were separately removed into 2 ml GC-MS vials, and dried in a Speed-Vac concentrator (model SVC 100H, Savant, NY).

The samples were then methoximylated and silylated. For methoximation, 50 μl of 20 mg/mL methoxyamine hydrochloride dissolved in dry pyridine was added. The reaction mixture was shaken at 30°C for 1.5 h. Silylation was performed by adding 70 μL of *N, O*-Bis (trimethylsilyl) trifluoroacetamide (BSTFA) with 1% trimethylchlorosilane (TMCS) and incubating at 65°C for 30 min.

One microliter of the derivatized samples was injected into the GC-MS in splitless mode. GC-MS analysis was performed using an Agilent 6890 GC interfaced to an Agilent 5973 quadrupole MS with a HP-5ms (5%-Phenyl)-methylpolysiloxane column (30 m × 0.25 mm × 0.25 μm, Agilent). The temperature was programmed from 70 to 320°C at 5°C/min with helium flow rate at 1.0 mL/min and inlet temperature at 280°C. EI-MS ionization energy was set to 70 eV and the interface temperature was 280°C.

The GC-MS data files were deconvoluted and searched against an in-house MS-library, the NIST 14 Mass Spectral Library using NIST AMDIS software (Stein, 1999) and the Golm Metabolome Database (Hummel et al., 2010).

Non-targeted metabolite profiling data obtained by GC-MS analysis, and anthocyanin data obtained from LC-fluorescence are publicly available in the PMR database (http://metnetdb.org/PMR/; Hur et al., 2013). They are available at: metnetweb.gdcb.iastate.edu/PMR/experiments/?expid=279.

### ELDI-MS

A Waters Synapt G2-S quadrupole time-of-flight mass spectrometer was used for data collection for all MSI. The Waters ESI source was removed and a homemade open-air ESI source (Galayda, 2017) was used with samples at ambient pressure. Data were acquired in the mass range from *m/z* 50 to 1200, spectra were summed for 0.3 s. The time-of-flight reflectron operated in single-pass mode with a resolution of ~10,000 FWHM for MS images. The TOF was then operated in double-pass mode with a resolution of ~40,000 FWHM, or “high resolution mode,” to confirm compound identifications by accurate *m/z* measurements. These confirmatory measurements were done on a different segment taken from the same leaf. Tandem MS quadrupole resolution varied from Δm = 5 to 12 depending on the analyte with a nominal collision energy of 20 eV. The Synapt was operated using Waters MassLynx V4.1 (SCN851) software.

### Data handling

Spectra were generated from total ion chromatograms (TIC) combined by the MassLynx software. The “.raw” Waters data files were converted to mzML files by Proteowizard Mass Converter Tool. The mzML files were then combined into an imzML file using imzML Converter. This combined image file was then viewed and images were generated from MSiReader V0.06 via the W.M. Keck FTMS Laboratory. All images made within MSiReader had Linear^2^ interpolation for image clarity, and used the “Jet” colormap/false color appearance. Co-localization and 3D images were created with MSiReader V1.00. For a few samples in the early stages of this study, images were generated after normalizing individual ion signals to the total ion signal. This normalization did not affect the images noticeably, so normalization was not used for any of the images shown below.

### ELDI compound identification protocol

Compounds were identified from the ELDI spectra in a multiple step process to ensure confident assignments. Initial ELDI measurements were done in so-called “sensitivity” mode, m/Δm ~10,000 FWHM. Based on these results, high resolution spectra at ~40,000 were acquired on a different segment of the same leaf for accurate mass measurements. Next, if the compound was of high enough abundance and without background interferences, tandem mass spectra were taken to provide a fragmentation pattern. These high resolution *m/z* values were then put into online metabolite databases (i.e., METLIN) to generate possible compound matches based on accurate mass and tandem MS (if available from both ELDI data and the online database). ELDI data were also compared to the corresponding GC-MS and LC-MS data acquired from Coleus Henna leaves for identity overlap. Every possible ID was given a Δppm value based on the experimental *m/z* value compared with the true *m/z* value of the compound. These Δppm values were generally below 10 ppm. Some compounds were given identifications with greater than 10 Δppm values based on matches with the GC-MS data and/or tandem MS matches with the online database. Some flavonoid peaks were assigned as cyanidin or apigenin derivatives despite no database matches due to highly abundant tandem MS product peaks at *m/z* 287.0585 or 271.0627, respectively. Many METLIN database entries of flavonoids did not have supporting MS/MS data for comparison, with some having only predicted MS/MS spectra.

Acquiring tandem MS data with ELDI was successful for most peaks of interest, but was not always possible. It was difficult to acquire tandem MS spectra for highly spatially localized compounds, labeled “localized signal” in subsequent data tables. Compounds with a relatively low abundance did not give a satisfactory fragmentation pattern for identification purposes (labeled “too low abun.” in subsequent data tables). Analyte peaks near major background peaks had interferences with their tandem MS spectra. The quadrupole resolution could sometimes be increased to eliminate these interferences, but a subsequent drop in peak intensity sometimes resulted in poor tandem MS results.

### ELDI source

The apparatus has been described (Galayda, 2017). Samples were ablated with a Nd:YAG laser (ULTRA, Big Sky Laser Tech, Inc. Bozeman, MT). The third harmonic was used at 355 nm. The laser was operated at a pulse repetition rate of 10 Hz, with a 5 ns pulse width, and an energy of ~250 μJ/pulse (before focusing). This pulse energy was just above the ablation threshold for these samples. The beam was focused onto the sample by a single plano-convex focusing lens (fused-silica, focal length 75 mm), with a nominal spot size of ~125 μm.

Samples were cut with a scalpel. Immediately after cutting, the sample segments were mounted on a glass slide using double sided tape. The laser did not penetrate completely through the sample; the underlying tape is not ablated. No matrix was applied, and leaves were pressed lightly to create an even surface. The optical images shown below were photographed before this pressing step. Thus, the dimensions of the optical images are slightly smaller than those of the MS images in some of the results shown below.

Plant samples were then placed 8 mm below the ESI-sample inlet axis on a computer-controlled translation stage (Z825B, Thorlabs, Inc. Newton, NJ). Samples were translated at 0.4 mm/s beneath the 10 Hz laser beam down the surface of the leaf to insure fresh tissue was constantly being ablated. The distance between the centers of adjacent ablation tracks was 125 μm, providing a lateral resolution of 125 μm. The ablation trench was ~ 30 μm deep. The underlying tape was not ablated. Mass spectra were averaged over 0.3 s intervals. Thus, the ablated volume was 125 × 120 × 30 μm deep. The leaves were irradiated normal to the sample surface with the laser beam axis ~2 mm downstream from the ESI capillary. The ESI tip was ~10 mm from the sample inlet (Galayda, 2017).

A solution of 50% methanol with 0.1% formic acid (99.5% purity, Fisher Scientific) was pumped through a 53 μm ID polyimide coated capillary as the ESI solution (pump: model Z2, Harvard Apparatus, South Natick, MA). Leucine enkephalin was added at 0.1 ppm to the ESI solution for use as a mass calibrant. All data were acquired in positive ESI mode. The ESI voltage for ELDI was +2.5 kV applied to a stainless steel union in the liquid flow line, with the sample cone completing the ESI circuit. The sample inlet was kept at 100°C with a N_2_ curtain gas flow of 1 L/h.

## Results

### Overview of experimental workflow

**Figure 1:**
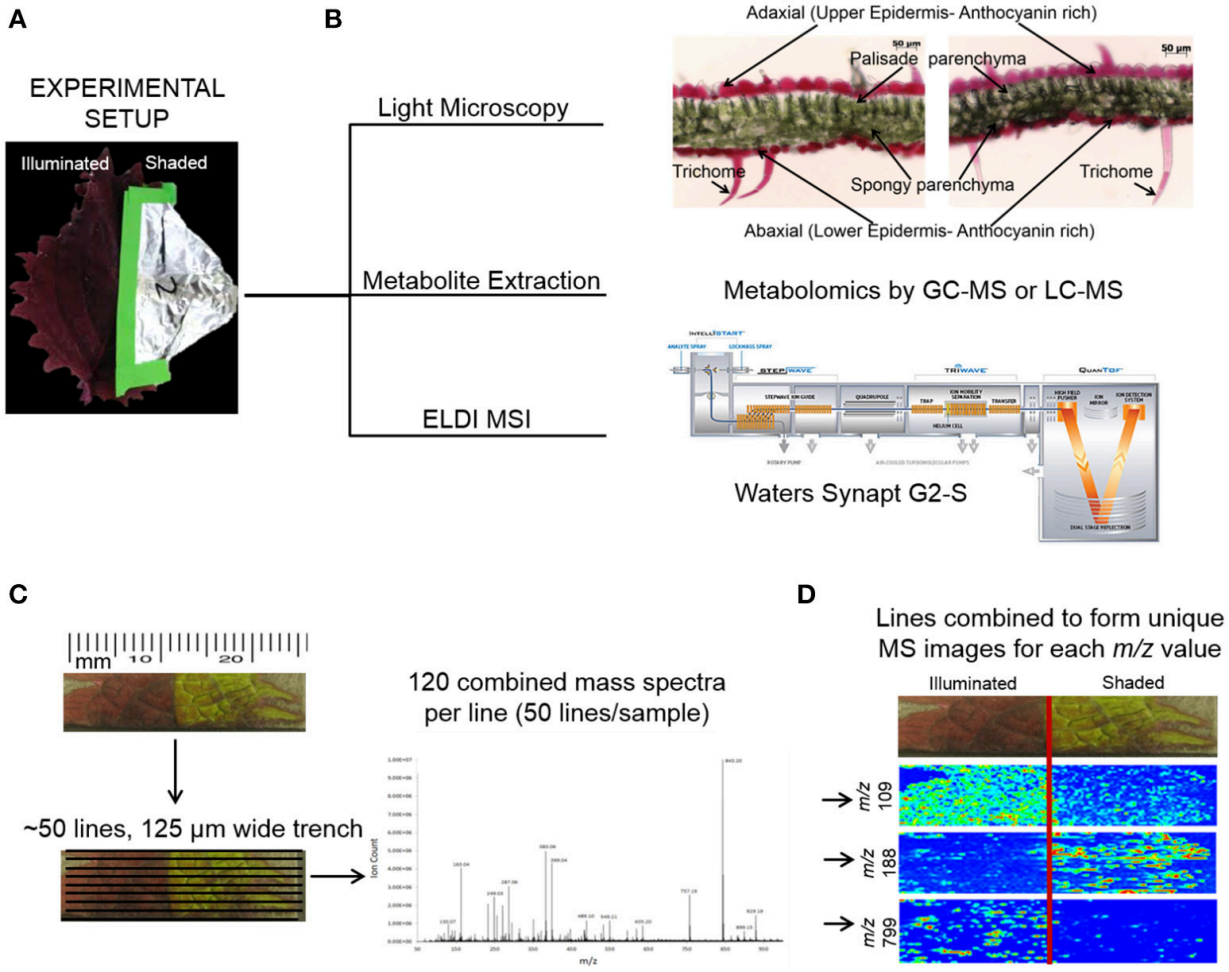
ELDI-MSI experimental workflow. **(A)** Individual Coleus Henna leaves were treated for a period of up to 28 days by shading half the leaf and leaving the other half fully illuminated. **(B)** Leaf samples were selected for light microscopy, metabolite extraction and metabolomics analysis, and ELDI-MSI analyses with a Waters Synapt G2-S mass spectrometer. **(C)** ELDI-MSI data were generated and validated by metabolite profiling of extracts by GC-MS or LC-MS analysis or METLIN database. **(D)** Mass spectrometric images were generated for individual ions and aligned with visual images of leaves.

The mid-rib (Figure 1) was used as the boundary between the two halves of each Coleus Henna leaf. One half of a leaf was covered with aluminum foil to generate leaf tissue that was grown under lower illumination conditions, and the uncovered half of the leaf was used as a control for normal levels of illumination. Each leaf was dissected transversely. Segments ~12 mm × 8 mm were collected from both the illuminated and shaded sides of the leaf. These segments were placed on a glass slide and used in ELDI imaging experiments.

Metabolite distribution maps were created for 77 chemically identified ions detected by ELDI-MS. Thirteen of these identified ions were observed to decrease in abundance in the shaded side of the leaf, while the abundances of 11 compounds increased in the shaded side. The relative abundances of the remaining 53 ions were unaffected by shading (Tables 1–3). In-parallel, targeted anthocyanin analysis via LC-MS, and non-targeted global metabolomic analysis via GC-MS were performed on separate leaf samples, which were sampled in triplicate.

**Table 1:** Flavonoids identified by ELDI-MS.

**High Res. *m/z***	**Assigned ID**	**Δppm of ID**	**Response to shading**	**Major fragment ions**	**Signal intensity**
255.0679	Catechine + H^+^−2H_2_O	6	None	Background interferences	5e4
271.0627	^*^Apigenin + H^+^	9	None	243, 229, 225, 156, 145, 119	1e6
287.0585	^*^Cyanidin (M^+^)	12	None	269, 241, 213, 185, 157, 137	7e5
291.0899	Catechine + H^+^	12	None	Too low abun.	2e4
399.0434	^*^Flavone + K^+^	10	None	381, 371, 353, 337, 287, 219, 201, 175	1e6
441.0725	Cyanidin Flavonoid + (M^+^) +H^+^	3	None days 7-14, decreased days 21-28	287	2e5
447.0918	^*^Apigenin Glucuronide + H^+^	0.4	None	271, 163	7e5
458.0823	Flavone + 2H^2+^	4	None	399, 371, 353, 219, 163, 147	8e4
476.0907	Cyanidin-coumaroyl glucoside-dimalonylglucoside (M^+^) + Na^+^	6	Decreased	287	7e4
483.0781	Cyanidin flavonoid (M^+^)		None	287, 203, 163, 147	8e5
489.1057	Apigenin flavonoid + H^+^ OR + H^+^ - H_2_O	4	None days 7-21, decreased day 28	471, 399, 271, 163	5e5
505.1016	Flavone glucoside + H^+^- H_2_O	6	None		5e4
511.0744	Cyanidin flavonoid (M^+^)		Decreased	437, 353, 313, 287, 271, 179	7e4
527.0515	Cyanidin flavonoid (M^+^)	3	Decreased	369, 309, 287, 163	1e5
533.2350	Flavonoid		None	287, 271, 219, 201, 163, 147	2e5
535.1069	^*^Cyanidin malonylglucoside (M^+^)	2	None days 7-21, decreased day 28	287, 163	6e4
549.2095	Cyanidin Flavonoid (M^+^)		None	369, 331, 287, 271, 219, 201, 163, 147	2e5
557.0940	Flavonoid + Na^+^	6	None	Interference from lock mass	8e4
593.1339	Flavonoid + H^+^- H_2_O	7	None	Too low abun.	2e4
595.1447	^*^Cyanidin coumaroylglucoside (M^+^)	0.1	Decreased	549, 287, 163, 147	3e5
619.2228	Apigenin flavonoid + H^+^		Increased	575, 533, 271, 163	2e5
635.2041	Apigenin flavonoid + H^+^		Increased	591, 549, 271, 163	4e5
697.1607	Flavonoid + H^+^	0.4	Decreased	Too low abun.	1e4
743.1454	Apigenin flavonoid + H^+^		None	271, 163, 147	4e4
757.1965	^*^^+^Cyanidin-coumaroylglucoside-glucoside (M^+^)	1	None	595, 449, 399, 287, 271, 163, 147	8e5
773.1890	Flavonoid + H^+^	4	None	Too low abun.	1e4
787.2041	Flavonoid + H^+^	4	None	Too low abun.	2e4
799.2031	Cyanidin flavonoid M^+^- H_2_O or M^+^- 2H_2_O	0.1–6	Decreased	621, 595, 535, 527, 441, 287, 163, 147	7e4
813.1613	Cyanidin flavonoid (M^+^)		None	795, 287, 163	7e4
827.1994	Flavonoid + H^+^	4-5	Decreased	Too low abun.	2e4
829.1490	Cyanidin flavonoid (M^+^)		None	595, 287, 271, 163, 147	5e4
843.1970	^*^^+^Cyanidin-coumaroyl glucoside- malonylglucoside (M^+^)	0.4	Decreased	799, 595, 535, 287, 163, 147	4e6
859.1876	^^*^+^Cyanidin- caffeoylglucoside-malonylglucoside (M^+^)	5	Decreased	843, 489, 287, 271, 163	7e4
865.1617	Flavonoid + H^+^- H_2_O	0.1–7	Decreased	839, 821, 677, 635, 617, 575, 531, 513	1e5
873.2090	Cyanidin flavonoid (M^+^)	0.6+	None	843, 693, 595, 535, 489, 287, 163, 147	5e4
883.1759	Dicatechine flavonoid + H^+^	4	None	865, 839, 821, 677, 635, 617, 575, 531, 513	2e5
885.2034	Flavonoid		Decreased	867, 595, 577, 287, 271, 163, 147	9e4
899.1660	Dicatechine flavonoid + H^+^	0.5	None	881, 855, 837, 813, 693, 651, 633, 591, 547	3e5
929.1966	^*^^+^Cyanidin-coumaroyl glucoside-dimalonylglucoside (M^+^)	1	Decreased	843, 621, 595, 489, 287, 163, 147	1e6
951.1619	Cyanidin flavonoid (M^+^)		Decreased	865, 843, 741, 635, 549, 489, 447, 399, 287	5e4

*Peak assignments were based on matches with entries in the METLIN Database (Smith et al., 2005)*.

* *denotes MS/MS peak matches with METLIN Database*.

+ *denotes metabolites identified through LCMS*.

**Table 2:** Carbohydrates identified by ELDI-MS.

**High Res. *m/z***	**Assigned ID**	**Δppm of ID**	**Response to shading**	**Major fragment ions**	**Signal intensity**
159.0021	Tetrose + K^+^	20	None	Localized signal	8e4
189.0158	^#^Pentose + K^+^	0.1	None	Localized signal	3e4
197.0433	^*^Organic Acid Glucoside + H^+^ + Na^+^	0.01	None	161, 145, 119, 101	1e6
203.0560	^*^^#^Hexose (glucose) + Na^+^	16	No abundance day 7, increased days 14-28	185, 167, 157, 137, 123, 111	1e3 - 5e5
219.0310	^*^^#^Hexose (glucose) + K^+^	20	No abundance day 7, increased days 14-28	201, 183, 151, 123, 111	5e3 - 6e5
233.0671	^*^Heptose + Na^+^	16	None	215, 205, 179	5e5
249.0414	^*^Heptose + K^+^	17	None	231, 213, 195, 125	4e5
255.1064	Hexose-glycerol + H^+^	4	None	Too low abun.	1e4
277.0901	Hexose-glycerol + Na^+^	0.1	None	Background interferences	5e4
312.9720	Hexose-Phosphate + K^+^	0.01	Low abundance day 7, increased days 14-28		3e3 – 9e4
337.0585	^*^Caffeoylglucarate + H^+^−2H_2_O	5	None	319, 185, 163	7e4
349.0923	^*^Organic Acid Glucoside + Na^+^	8	Increased	331, 313, 267, 163, 149	9e4
357.0586	Hexose-glycerol Phosphate + Na^+^	8	None, decreased in abundance day 28		4e3 – 1e5
365.0647	^*^Organic Acid Glucoside + K^+^	3	Increased	347, 333, 329, 163	1e5
365.1064	^*^^#^Sucrose + Na^+^	2	None	347, 337, 319, 203	5e4
371.0475	^*^Galloylglucose + K^+^	26	None	353, 327, 237, 219	1e5
381.0786	^*^^#^Sucrose + K^+^	2	None	363, 345, 335, 219, 163, 147	7e4

*Peak assignments were based on matches with entries in the METLIN Database (Smith et al., 2005)*.

* *denotes MS/MS peak matches with METLIN Database*.

# *denotes metabolites identified through GCMS*.

**Table 3:** Amino acids and organic acids identified by ELDI-MS.

**High Res *m/z***	**Assigned ID**	**Δppm of ID**	**Response to shading**	**Major fragment ions**	**Signal intensity**
72.0454	Alanine + H^+^ – H_2_O	6	None	Too low *m/z*	7e3
73.0309	^#^Lactic Acid + H^+^ – H_2_O	26	None	Too low *m/z*	1e4
81.0707	Caproic Acid + H^+^ – 2H_2_O	3	None	Too low *m/z*	1e4
83.0145	^#^Succinic Acid + H^+^ – 2H_2_O	7	None	Too low *m/z*	8e3
86.0588	GABA + H^+^ – H_2_O	20	None	Too low *m/z*	3e4
98.0198	^#^Glycine + Na^+^	14	None	Too low abun.	6e3
98.0585	Proline H^+^ – H_2_O	21	None	Too low abun.	9e3
106.0494	^*^^#^Serine + H^+^	4	None	88	1e4
114.0907	^#^Leucine + H^+^ – H_2_O	10	None	Background interferences	1e3 – 2e5
115.0029	^#^Tartaric Acid + H^+^ – 2H_2_O	6	None	Localized Signal	2e4
116.0365	^*^Aspartic Acid + H^+^ – H_2_O	14	Increased	88	3e4
118.0863	^*^Valine + H^+^	~0	None	100, 72	3e4
120.0658	^*^Threonine + H^+^	2	None	102	6e4
129.0346	^*^^#^p-Coumaric Acid + H^+^-2H_2_O	~0	None	119, 91	2e4
130.0520	^*^^#^Pyroglutamic Acid + H^+^	16	None	84	4e5
145.0322	^*^^#^Caffeic Acid + H^+^ – 2H_2_O	18	None	135, 117, 107, 89, 79	1e5
147.0485	^*^^#^p-Coumaric Acid + H^+^-H_2_O	26	None	119, 91	4e5
147.0777	^*^Glutamine + H^+^	8	None days 7-21, Increased day 28	130, 84	2e4
155.0423	^*^Asparagine + Na^+^	2	None	109	2e4
156.0809	^*^Histidine + H^+^	26	None days 7-14, Increased days 21-28	110, 93, 83	5e4
157.0133	^#^Citric Acid + H^+^ – 2H_2_O	6	None	Too low abun.	9e3
163.0430	^*^^#^Caffeic Acid + H^+^-H_2_O	20	None	145, 135, 117, 107, 89, 79	8e5
170.0563	^*^^#^Leucine + K^+^	8	None	124	5e4
175.1200	^*^Arginine + H^+^	5	Increased	158, 130, 116, 112, 70	1e5
219.0019	^*^^#^Caffeic Acid + K^+^	16	None	201, 183, 173, 161, 129	3e5
230.9883	^*^^#^Citric Acid + K^+^	6	None	213, 195	2e4

*Peak assignments were based on matches with entries in the METLIN Database (Smith et al., 2005)*.

* *denotes MS/MS peak matches with METLIN Database*.

# *denotes metabolites identified through GCMS*.

### Illumination affects foliage color and anthocyanin abundances

**Figure 2:**
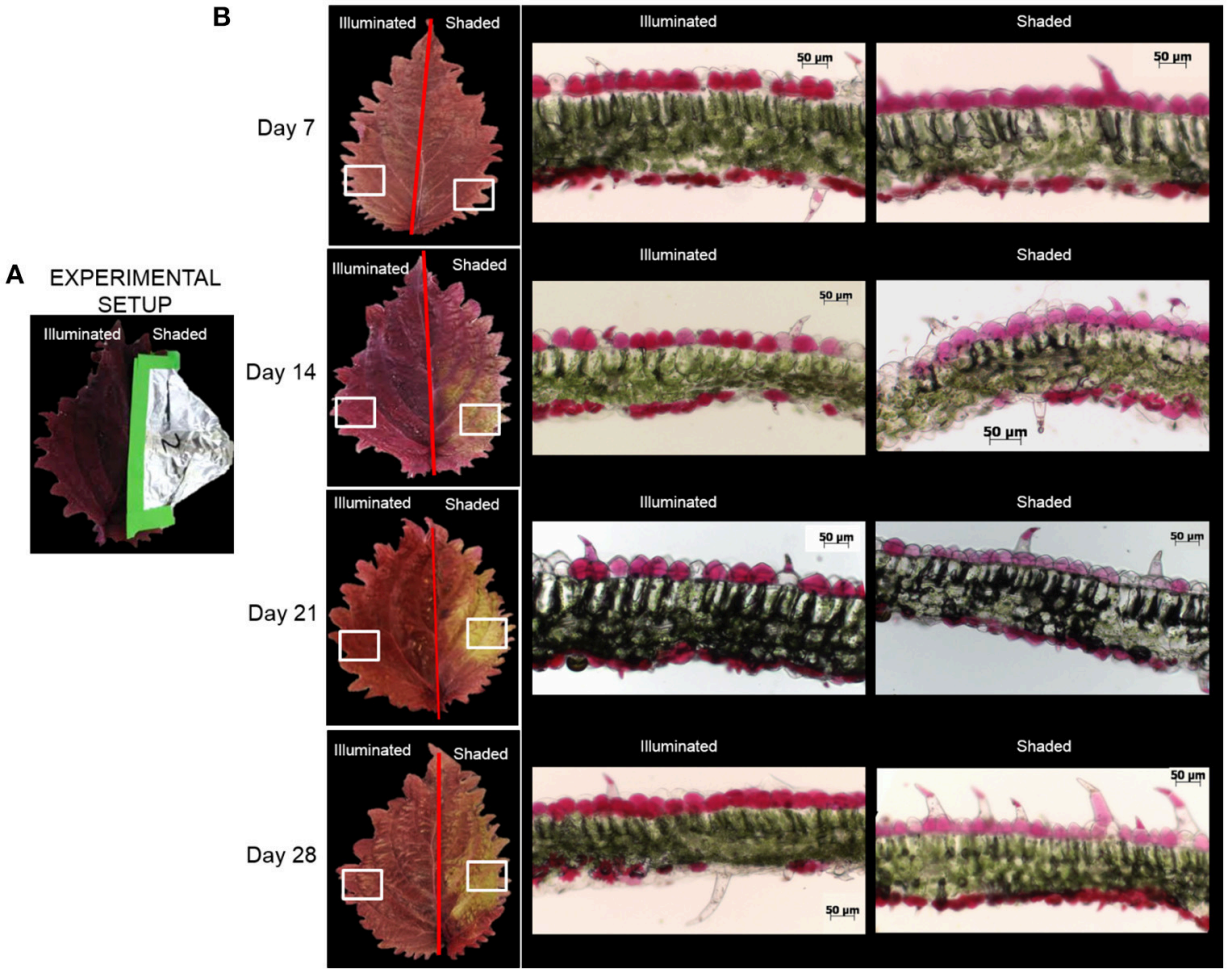
Optical images of Coleus Henna leaves over a 28 day period. **(A)** The right-side of each leaf was shaded by wrapping with aluminum foil, whereas the left side was fully illuminated. White boxes denote areas of the leaves that were sectioned for optical microscopic examination in cross section **(B)**. Scale bar = 50 μm.

Under normal illumination conditions both the adaxial (upper) and abaxial (lower) surfaces of the Coleus Henna leaf are a burgundy red-purple color. In the shaded condition, the foliage color changes from burgundy red-purple to chartreuse green (Figure 2). Microscopic cross-sections of leaves reveal that the abaxial and adaxial epidermal cells are heavily red-pigmented, and the mesophyll and palisade layers are green pigmented with chlorophylls. Epidermal cells range from 20 to 50 μm deep with cross-sections in the range of 40 to 55 μm long. These cell dimensions are unaffected by shading. The red pigmentation was decreased in the adaxial epidermis, and unaltered in the abaxial epidermis. This loss of the red pigment from the adaxial epidermis revealed the underlying chlorophyll pigments, imparting the green color to the shaded half of the adaxial surface of the leaf.

### Effect of shading on the extractable leaf metabolome

**Figure 3:**
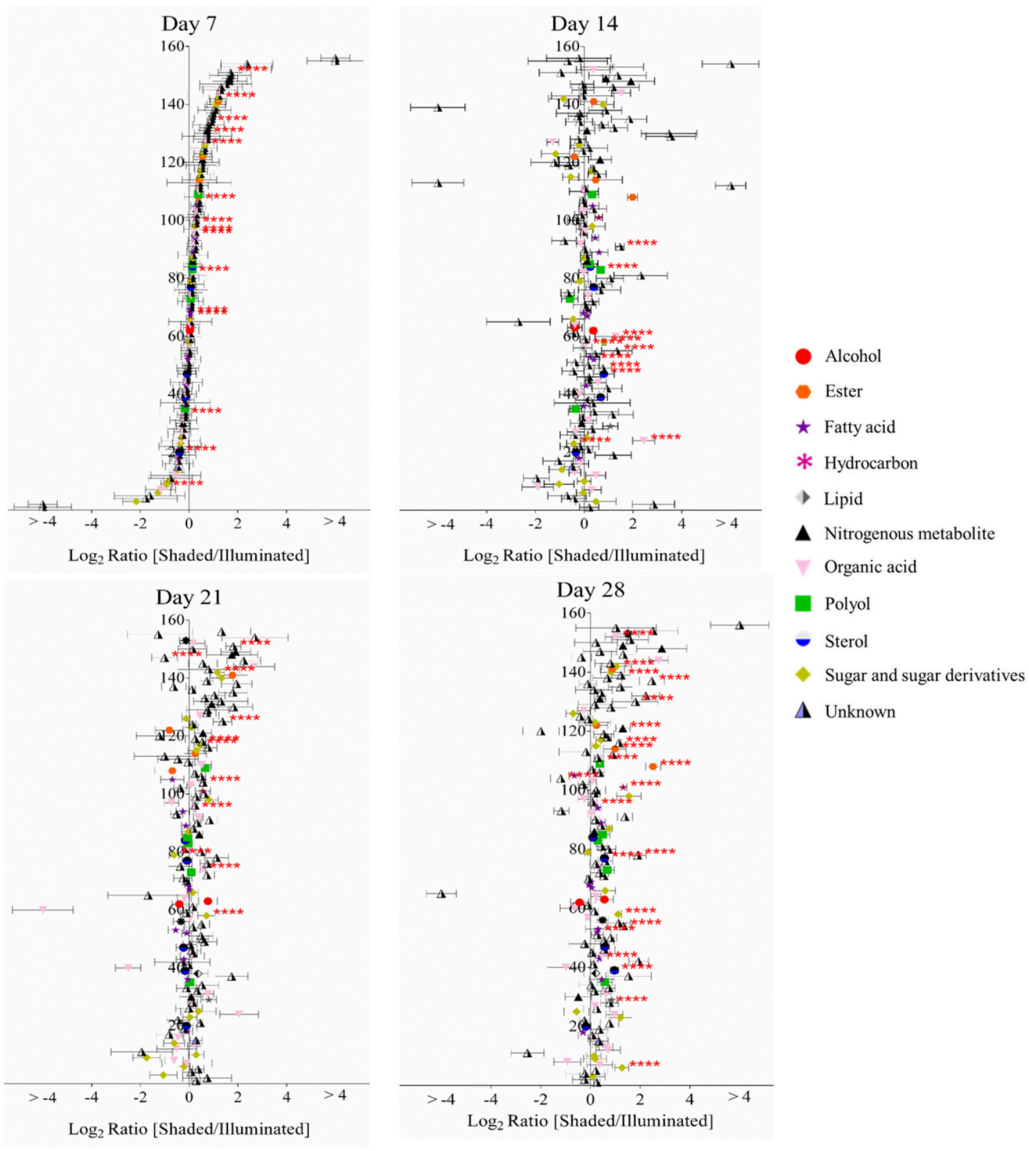
Log-ratio comparison of the differential metabolomes between illuminated and shaded sides of the leaf. The x-axis plots the log-transformed relative abundance ratio of each metabolite in illuminated versus shaded sides. The order of the metabolites (156 analytes, 67 chemically identified) on the y-axis is identical in all four plots, and they are ordered from the lowest to highest value on the x-axis as determined for the 7-day time-point. ^****^ denote *p*-value < 0.05.

The GC-MS analyses of metabolite extracts from leaf tissue detected 156 polar and non-polar analytes; 67 of these were identified chemically. These metabolites include alcohols, polyols, sugars, lipids, fatty acids, esters, sterols, hydrocarbons, organic acids, and nitrogenous metabolites. The abundances of most of the chemically identified metabolites remained unaltered irrespective of the illumination conditions (Figure 3). The abundances of a few metabolites, such as gluconic acid, fructose, arachidonic acid, 1-monopalmitin and a hexose sugar, increased in the shaded side of the leaf as time progressed. The abundance of fructose increased in the shaded side of the leaf and became higher than in the illuminated side by the 28th day of the experiment. Benzoic acid showed a unique profile, with abundance decreasing through the time-line of the experiment; its abundance was always higher in the shaded side of the leaf.

**Figure 4:**
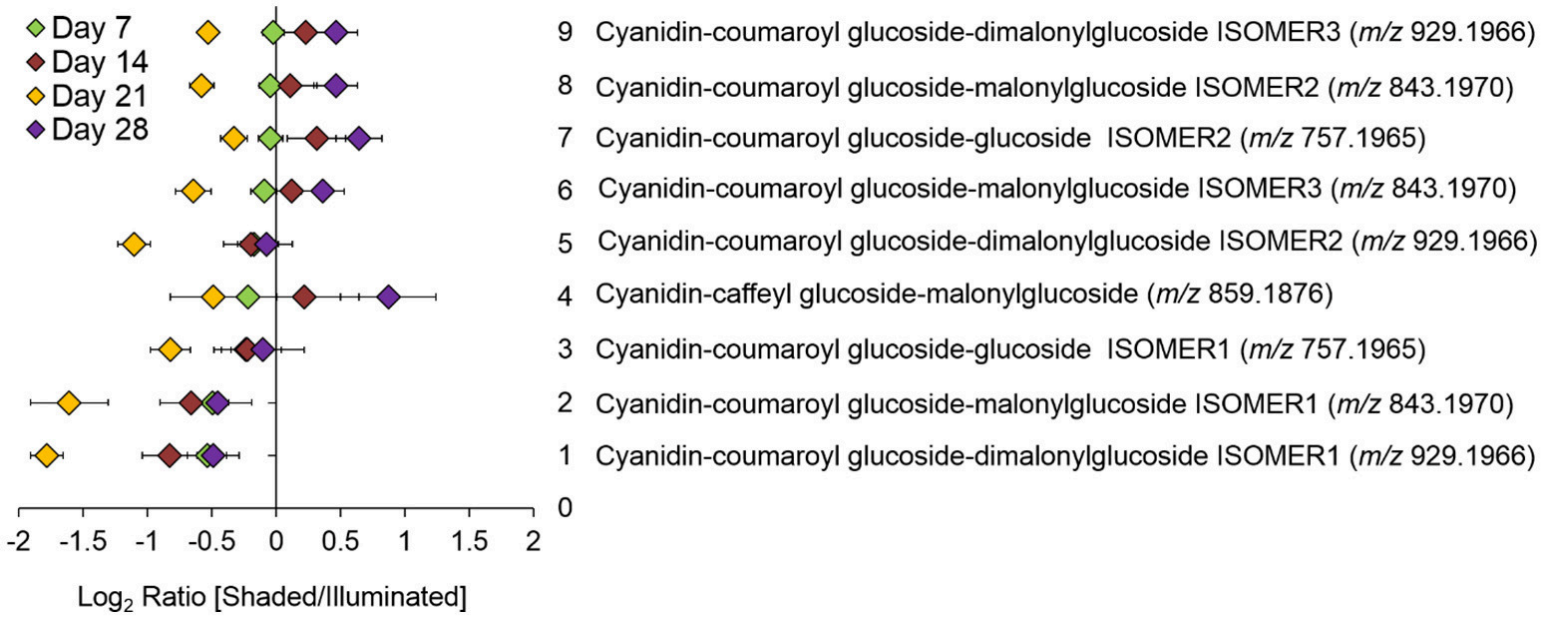
Log-ratio comparison of the differential accumulation of anthocyanins in Coleus Henna half-leaves maintained under full-illumination or shaded for the indicated time-periods. The table names each of the 9 anthocyanins that were identified by LC-MS analysis of extracts prepared from the two leaf halves. The order of the metabolites on the y-axis of the plot is from the lowest to the highest log-ratio values as determined for the 7-day time point.

Targeted LC-MS analyses measured changes in anthocyanin concentrations during these experiments. Nine cyanidin-based anthocyanins (Figure 4) were identified. Two of these showed differential accumulation between the illuminated and shaded sides of the leaf: cyanidin-coumaroylglucoside-malonylglucoside at *m/z* 843.1970 and cyanidin-coumaroylglucoside-dimalonylglucoside at *m/z* 929.1966. The abundance of both these anthocyanins decreased significantly in the shaded side of the leaf after 21 days of the experiment. Day 21 appears to be anomalous compared to the other time points. We suggest that day 21 may represent a metabolic switch, as the leaf is depleted of carbon and subsequently is re-accessed from sink tissues. The accumulation of the other 7 anthocyanins was unaffected by the difference in illumination.

### ELDI-MS identification of flavonoids

**Figure 5:**
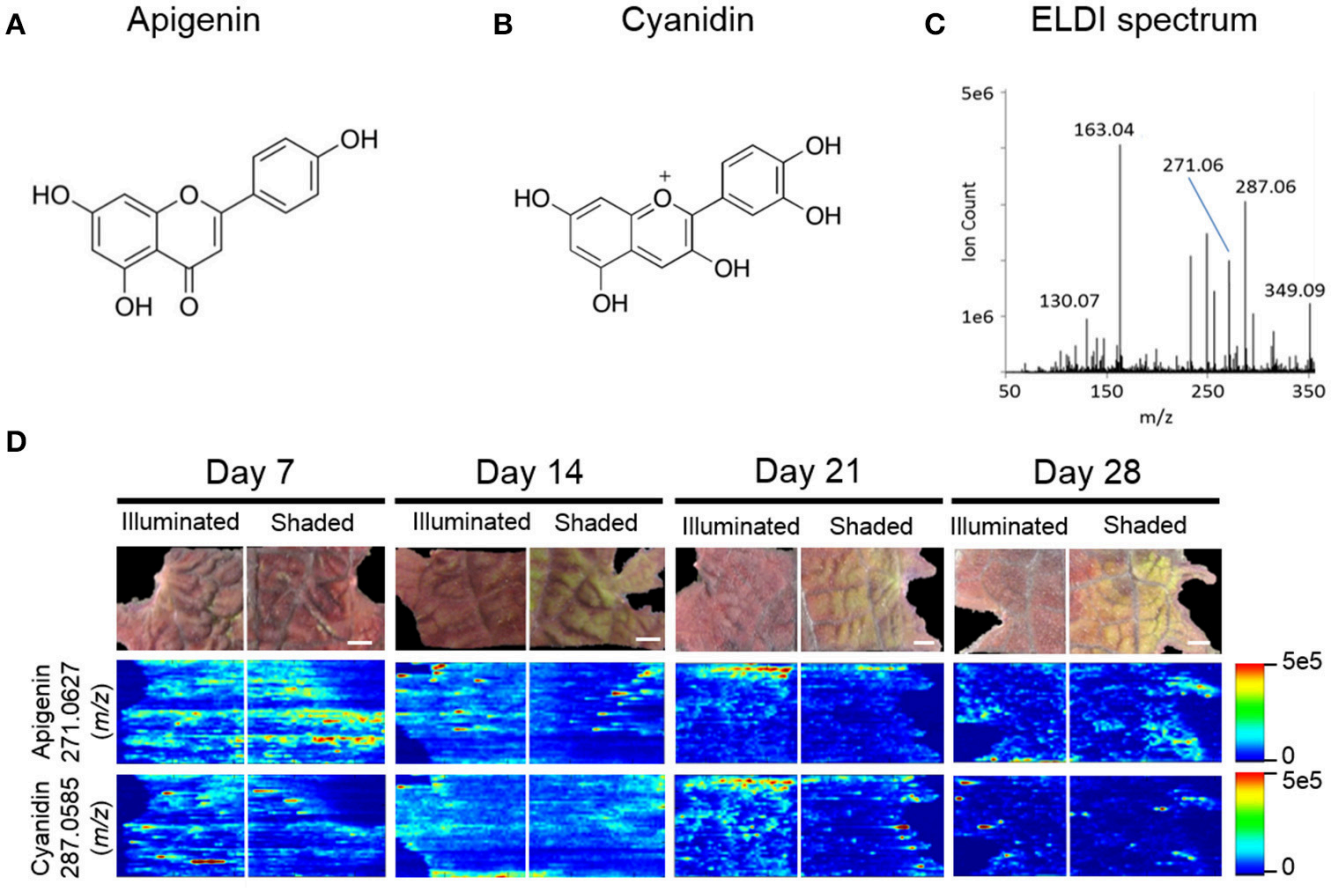
ELDI-MSI analysis of flavonoid backbones. Molecular structure of two aglycone flavonoids. **(A)** apigenin; and **(B)** cyanidin. **(C)** ELDI mass spectrum showing the relative abundance of apigenin ([M+H]^+^ at *m/z* 271.0627) and cyanidin ([M]^+^ at *m/z* 287.0585) ions. **(D)** Spatial distribution of apigenin and cyanidin in shaded or fully illuminated leaf-halves, at different time-points after initiation of the shading treatment. Scale bar = 2 mm. The MSI intensity scale bar is color coded: red is maximum signal and blue is minimum signal, in this and subsequent figures.

The pigmentation on the abaxial side of the leaf was unaffected by shading, whereas the pigmentation pattern was altered on the adaxial side of the leaf. Therefore, we focused on imaging the distribution of the metabolites on the adaxial surface using ELDI-MSI. Many of the ions detected in these ELDI spectra were attributed to flavonoids. Their chemical identity was confirmed by MS/MS experiments that generated fragment ions characteristic of the flavonoid backbones. The identities of these fragment ions were confirmed by matching accurate mass measurements with entries in the Metlin database (Smith et al., 2005). This strategy identified two main classes of flavonoids, based on the aglycone cores: (a) apigenin, a flavone (Figure 5A), and (b) cyanidin, an anthocyanin (Figure 5B).

Apigenin was identified as its protonated ion [M+H]^+^ at *m/z* 271.0627. All analytes that generated this fragment ion by tandem MS were inferred to be apigenin-based flavones. Cyanidin has a permanent +1 charge and was identified as an [M]^+^ ion at *m/z* 287.0585. Cyanidin was distinguished from isobaric compounds (e.g., luteolin or kaempferol) based on MS/MS fragments derived from this [M]^+^ ion (Table 1). Cyanidin-based anthocyanins were identified by cyanidin [M]^+^ product ions at *m/z* 287.0585. The high intensities of the apigenin-aglycone (*m/z* 271.0627) and cyanidin-aglycone (*m/z* 287.0585) ions in the overall ELDI spectrum (Figure 5C) show that the aglycones have a high relative abundance compared to other low mass molecules observed.

The cyanidin-based anthocyanins are primarily glycosylated and further biochemically modified by malonylation or coumarylation (Table 1). These anthocyanins generated common fragment ions at *m/z* 163.0620 and 147.0485, which were identified as the protonated water-loss ion [M-H_2_O+H]^+^ of a hexoglycoside, and the protonated water-loss ion [M-H_2_O+H]^+^ of coumaric acid, respectively. The glycone moiety was not identified directly. However, based on the METLIN and the KEGG pathway databases (Smith et al., 2005; Guijas et al., 2018), and prior characterization of these metabolites in other Coleus lines (Boldt, 2013), these glycosides are subsequently referred to as glucosides. Collectively therefore, we identified 15 cyanidin-based anthocyanins (Table 1).

Similar characterizations identified four apigenin-based flavones, including the apigenin aglycone. Tandem MS generated the apigenin backbone product ion, but the specific chemical structures of these apigenin-based flavones were not determined. The ELDI experiments identified five apigenin-based flavones at *m/z* 447.0918*, m/z* 489.1057*, m/z* 619.2228, m/z 635.2041, and *m/z* 743.1454 (Table 1).

### Effect of shading on the spatial distribution of flavonoids

**Figure 6:**
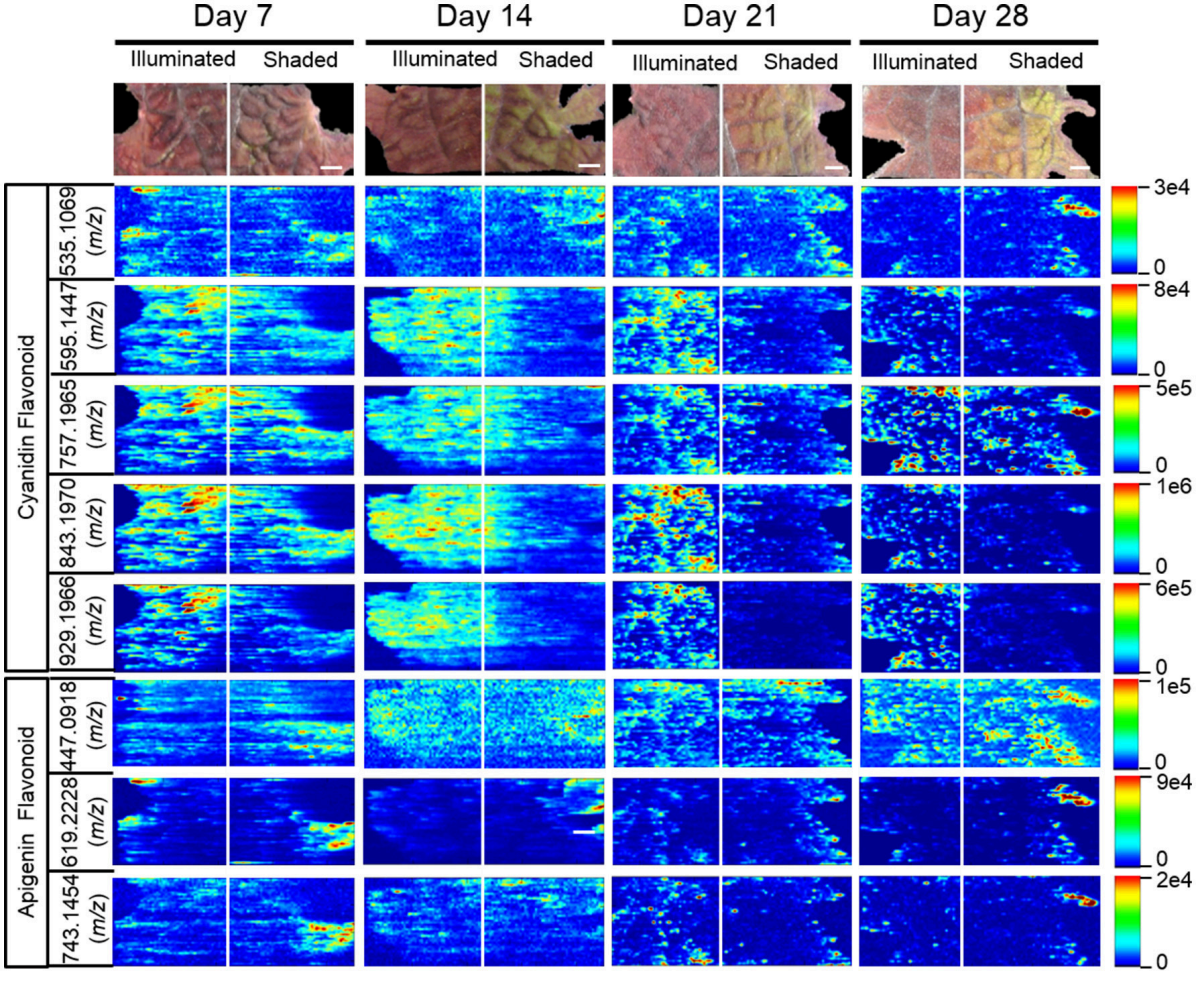
Spatial distribution between shaded and illuminated halves of leaves of cyanidin and apigenin-based flavonoids as affected by up to 28 days of shading. The cyanidin-based flavonoids were detected as [M]^+^ ions, and apigenin-based flavonoids were detected as [M+H]^+^ ions. The assigned IDs of the cyanidin-based flavonoids are as follows: cyanidin malonylglucoside (*m/z* 535.1069), cyanidin coumaroylglucoside (*m/z* 595.1447), cyanidin-coumaroylglucoside-glucoside (*m/z* 757.1965), cyanidin-coumaroyl glucoside- malonylglucoside (*m/z* 843.1970), cyanidin-coumaroyl glucoside-dimalonylglucoside (*m/z* 929.1966). The apigenin-based flavonoids are tentatively identified as apigenin glucuronide (*m/z* 447.0918), apigenin flavonoid (m/z 619.2228), and apigenin flavonoid (*m/z* 743.1454). Scale bar = 2 mm.

The 15 chemically identified cyanidin-derived anthocyanins can be categorized into three classes. Eight are less abundant in the shaded side of the leaves, one is more abundant in the shaded side, and six are not affected by the difference in illumination (Table 1). This last category includes the cyanidin aglycone, whose abundance are unaffected by shading during the entire 28-day period of the study, same as the apigenin aglycone (Figure 5D). Additional example images are presented in Figure 6, which shows the spatial distribution of five chemically identified cyanidin-based anthocyanins and three apigenin-based flavonoids. These include cyanidin-malonylglucoside (*m/z* 535.1069 [M]^+^), cyanidin-coumaroyl glucoside (*m/z* 595.1447 [M]^+^), cyanidin-coumaroylglucoside-glucoside (*m/z* 757.1965 [M]^+^), cyanidin-coumaroylglucoside-malonylglucoside (*m/z* 843.1970 [M]^+^), and cyanidin-coumaroylglucoside-dimalonylglucoside (*m/z* 929.1966 [M]^+^); and the apigenin flavonoids at *m/z* 447.0918 [M+H]^+^, *m/z* 619.2228 [M+H]^+^, and *m/z* 743.1454 [M+H]^+^.

MS images in this paper are obtained by scanning the laser horizontally from left to right. Signals are not acquired between adjacent ablation tracks. This acquisition method generates images that appear to be elongated horizontally, largely due to the way in which the data are acquired. Additional horizontal stretching could occur because (a) there is no delay between pixels to allow recently ablated material to wash out of the ion source, and (b) ablation occurs into room air; there is no controlled gas flow in the ion source. Two examples are the two laterally-elongated features seen in Figure 5D for apigenin, day 14 shaded side, middle of image. The effect is intermittent; it is not always observed. When this elongation is observed, as in Figure 5D, usually multiple adjacent tracks are elongated, which indicate that stretched images are mainly due to the stretched analyte regions in the actual sample.

**Figure 7:**
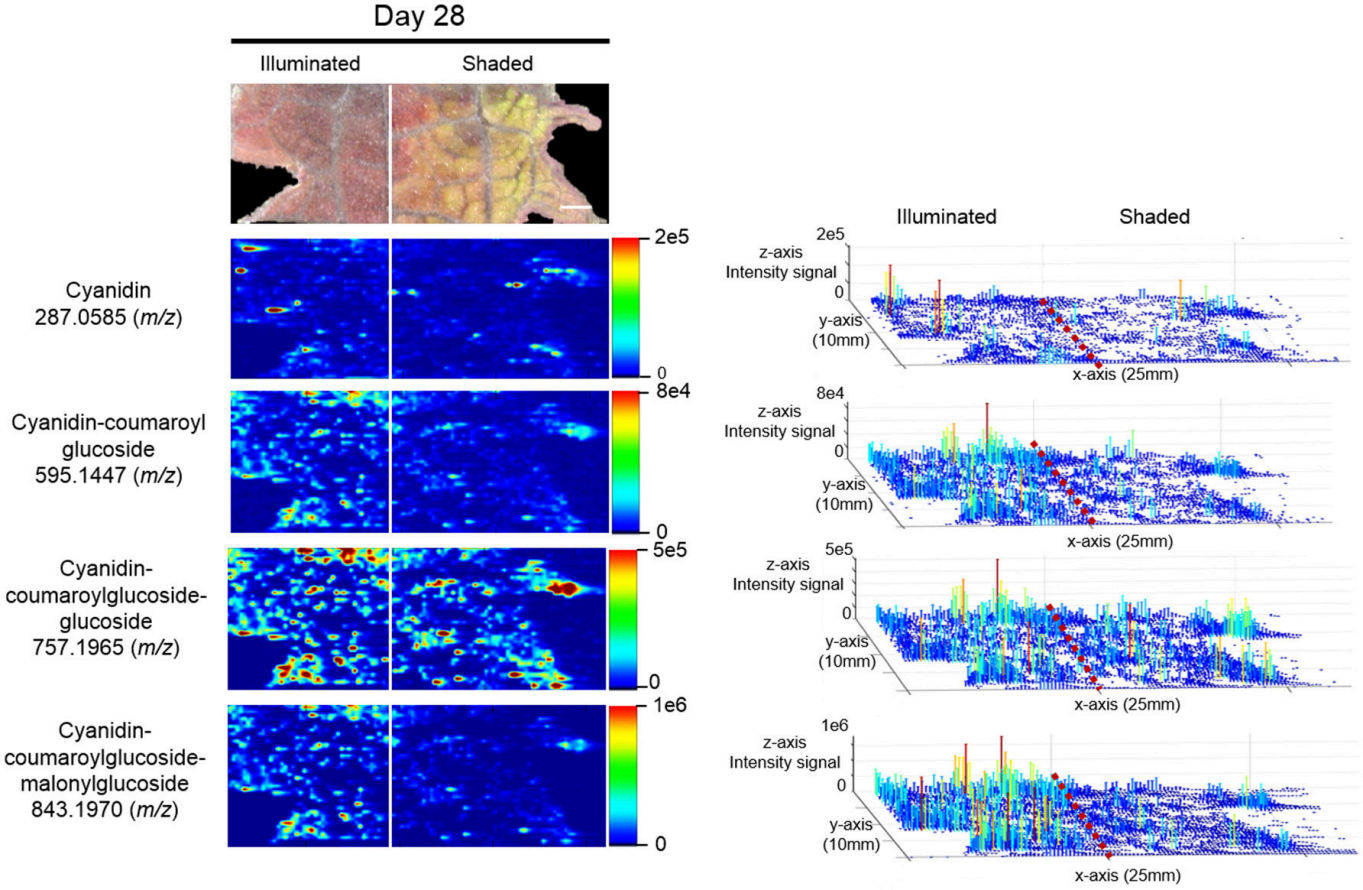
Spatial distributions of cyanidin-coumaroylglucoside-malonylglucoside and its metabolically related species determined by ELDI-MSI analysis of leaf-halves subjected to either full illumination or shaded for 28-days. Right panel represents the 3D representations of the abundance of these metabolically related ions. The x- and y-axes represent spatial coordinates (mm), and the z-axis maps ion intensity. Scale bar = 2 mm.

The anthocyanin with the highest relative abundance, cyanidin-coumaroylglucoside-malonylglucoside (*m/z* 843.1970 [M]^+^), is initially equally abundant in both illuminated and shaded sides of the leaf. However, starting at 14 days its abundance is reduced more rapidly in the shaded side of the leaf (Figure 6). A similar distribution pattern after 28 days of shading is observed for the structurally related cyanidin-coumaroylglucoside (*m/z* 595.1447 [M]^+^). In contrast, cyanidin-coumaroylglucoside-glucoside (*m/z* 757.1965 [M]^+^) is equally abundant in both the illuminated and shaded sides after 28 days (Figure 6). This anthocyanin had the second highest relative abundance and may be the immediate metabolic precursor to cyanidin-coumaroylglucoside-malonylglucoside (Guijas et al., 2018). Three-dimensional temporal distribution patterns of these structurally related anthocyanins (*m/z* 843.1970 [M]^+^, *m/z* 757.1965 [M]^+^, *m/z* 595.1447 [M]^+^, and *m/z* 287.0585 [M]^+^) after 28-days of shading are presented in Figure 7.

Similar to cyanidin-coumaroylglucoside-glucoside, the abundance of the dimalonylated derivative, cyanidin-coumaroylglucoside-dimalonylglucoside (*m/z* 929.1966 [M]^+^), is lower in the shaded side of the leaf (Figure 6). A precursor to cyanidin-coumaroylglucoside-dimalonylglucoside, the cyanidin-malonylglucoside (*m/z* 535.1069 [M]^+^) is equally abundant in both the illuminated and shaded sides until 28-days of shading, where its abundance is reduced in the illuminated side of the leaf (Figure 6). All these changes in relative abundance of cyanidin-based anthocyanins, revealed by ELDI-MSI, were confirmed by LC-MS analysis of extracts from these leaves (Figure 4).

The spatial redistribution of two apigenin-based metabolites was revealed by MSI. For example, the apigenin-based flavonoid at *m/z* 619.2228 occurs in both illuminated and shaded sides of the leaves and concentrates in the periphery of the shaded leaf-half (Figure 6). Another apigenin-based flavonoid at *m/z* 743.1454 is initially located in the periphery of the shaded leaf-half. It redistributes temporally to become more evenly dispersed among the two halves of the leaf (Figure 6).

### MSI profiles of carbohydrates

Twelve sugars and sugar derivatives were chemically identified using three MS-based criteria: (a) accurate mass determination with ELDI; (b) ELDI-MS/MS fragmentation spectra; and (c) integrated retention index and electron impact fragmentation patterns from GC-MS analysis. Most of the 12 sugars were observed as ^39^K^+^ and/or Na^+^ adducts by ELDI (Table 2). Specifically, sucrose was identified by MS/MS fragmentation patterns upon ELDI, and also by GC-MS analysis of extracts. A hexose, possibly glucose, was identified as both Na^+^ (*m/z* 203.0560) and ^39^K^+^ (*m/z* 219.0310) adducts. The identity of a phosphorylated hexose (possibly glucose-phosphate) was inferred from accurate mass determination of the ^39^K^+^ adduct (*m/z* 312.9720). An organic acid glucoside (possibly coumaroyl glucoside) was identified by MS/MS experiments as both the Na^+^ (*m/z* 349.0923) and ^39^K^+^ (365.0647) adducts. A heptose was identified by accurate mass determination and by MS/MS fragmentation spectra, but the particular isomer could not be determined.

**Figure 8:**
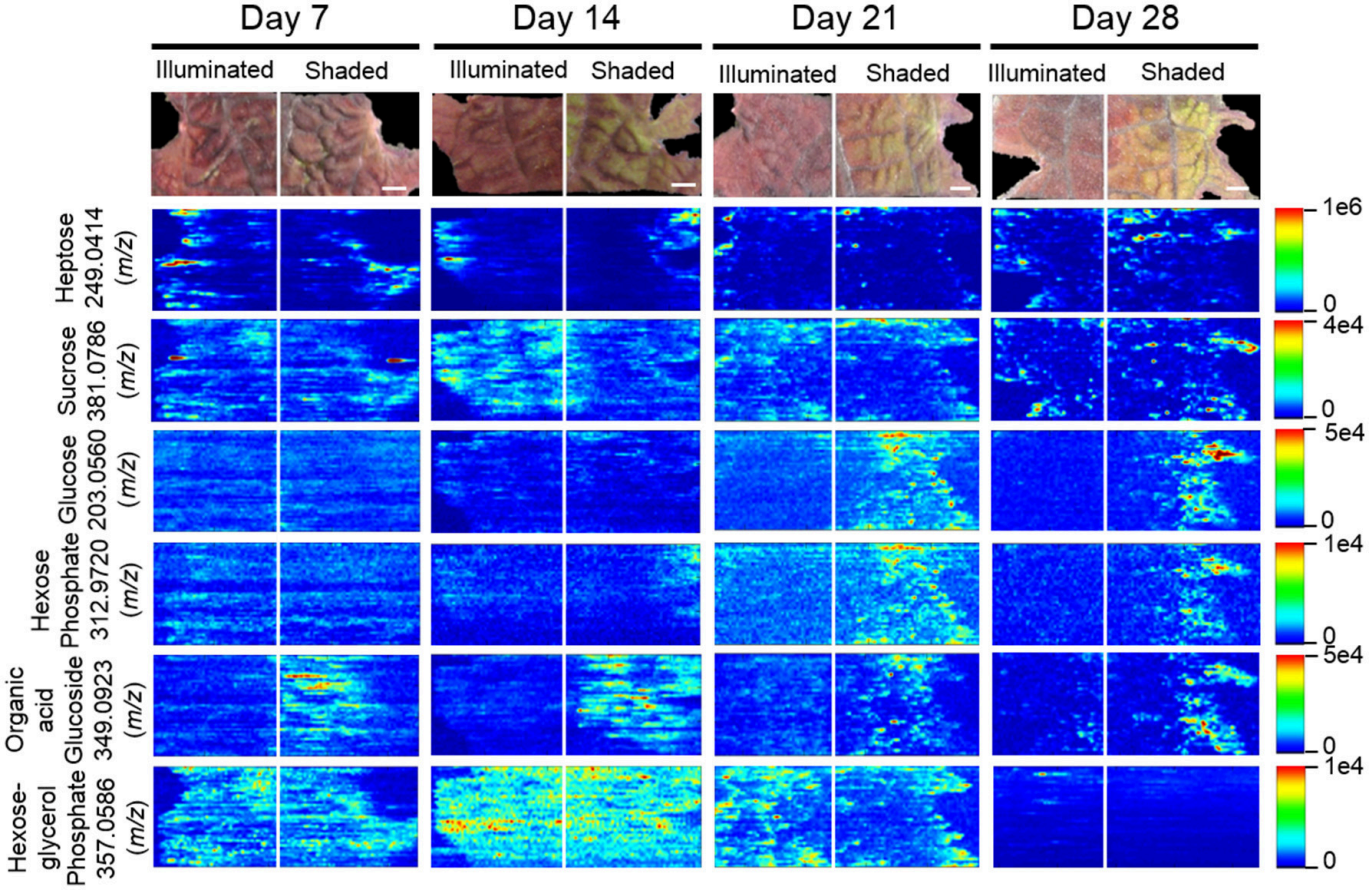
Spatial distributions of sugars determined by ELDI-MSI analysis of leaf-halves subjected to either full illumination or shading for up to 28-days. Each sugar was detected as [M+Na]^+^ or [M+K]^+^ adducts. Scale bar = 2 mm.

The abundances of eight of these sugar metabolites were unaffected by the illumination status of the leaf (Table 2). The spatial distributions of six typical metabolites are shown in Figure 8. Three of these sugar metabolites (*m/z* 203.0560, *m/z* 312.9720, *m/z* 349.0923) become more abundant over time in the shaded side of the leaf. Finally, a sugar-derivative, believed to be hexose-glycerol phosphate (*m/z* 357.0586), as determined by accurate mass, became less abundant across the entire leaf after 28 days of shading (Figure 8).

### MSI profiles of organic and amino acids

Table 3 lists the carboxylic acids and amino acids that were observed by ELDI-MSI. Seven of these metabolites were identified as H^+^ adducts, 15 as H^+^ adducts accompanied by water loss, two as Na^+^ adducts, and three as ^39^K^+^ adducts. Several of these compounds were observed as multiple adducts; individual molecules of a given compound had one of either H^+^, Na^+^ or ^39^K^+^ attached in the same spectrum.

**Figure 9:**
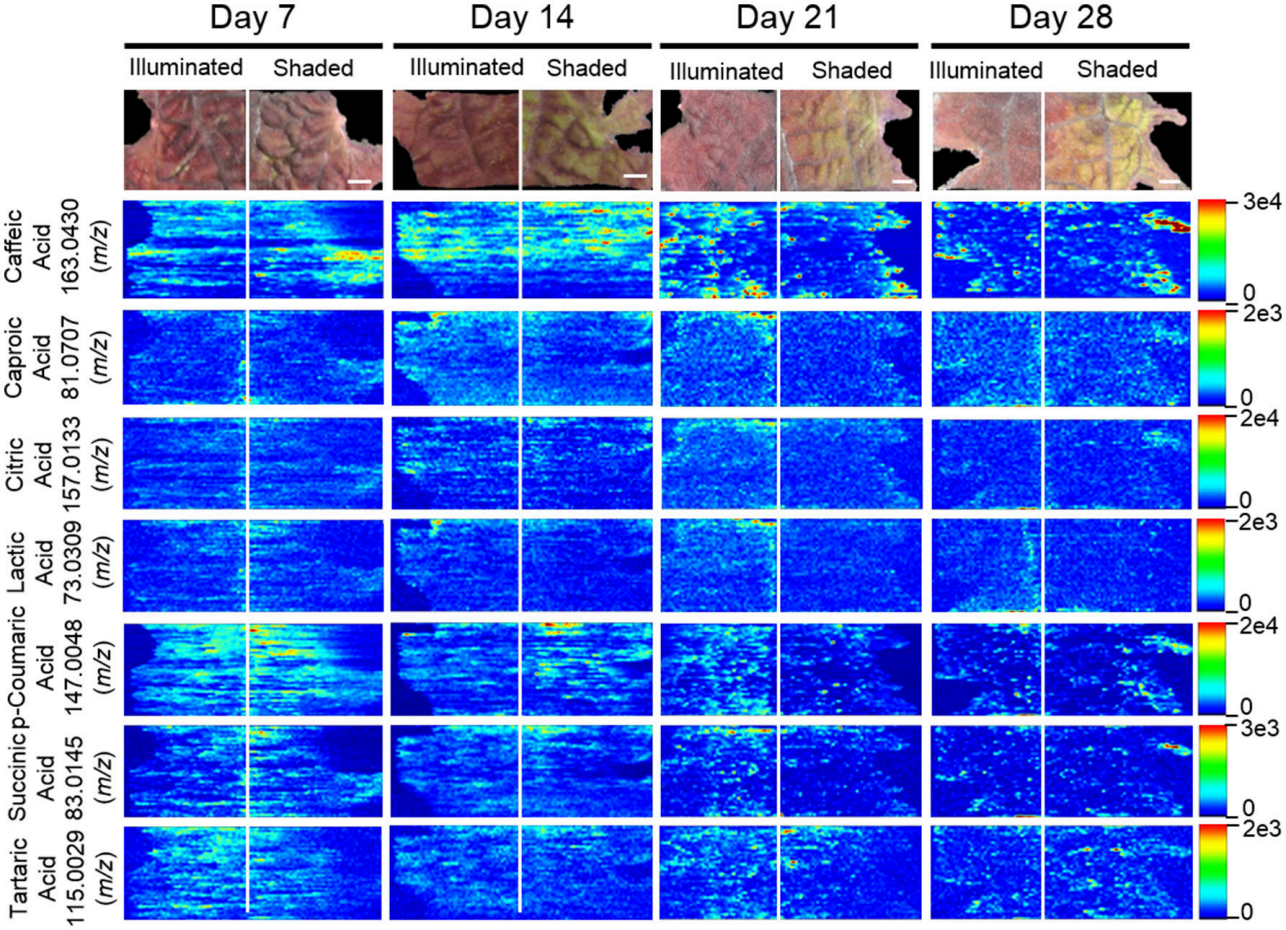
Spatial distributions of organic acids determined by ELDI-MSI analysis of leaf-halves subjected to either full illumination or shading for up to 28-days. Scale bars = 2 mm.

Several analytical strategies were integrated to confirm the chemical identity of these organic acids, including accurate mass determination, MS/MS experiments, and GC-MS analysis of derivatized metabolite extracts. The latter strategy was also used to confirm the relative concentrations between the illuminated and shaded sides of the leaves. Collectively these analyses identified 23 organic acids. Eight were identified with ELDI-MS and the molecular images of these metabolites (Figure 9) indicate their abundances were unaffected by the shading of the leaf, except for glycolic acid seen only by GC-MS.

**Figure 10:**
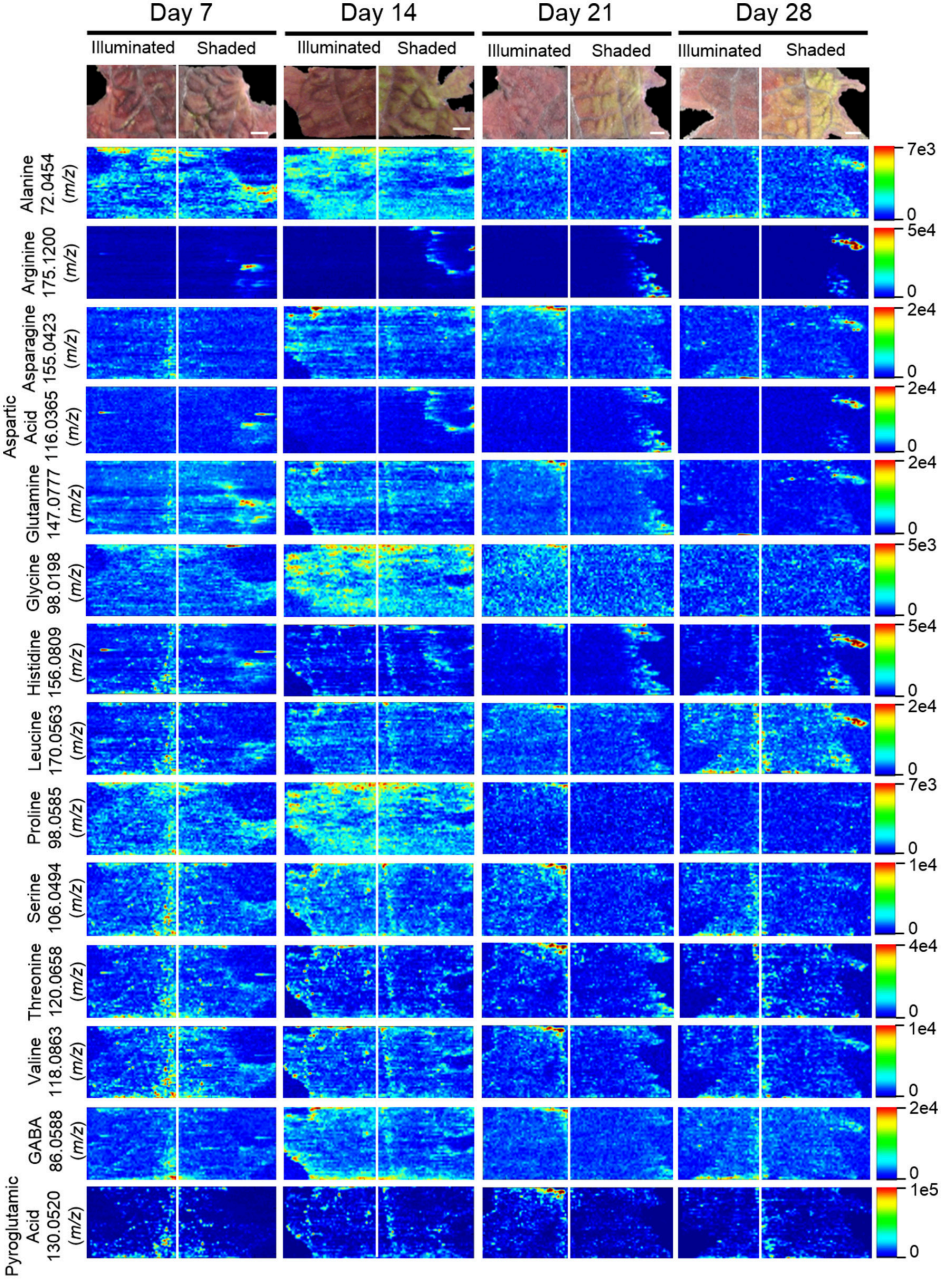
Spatial distribution of amino acids determined by ELDI-MSI analysis of leaf-halves subjected to either full illumination or shading for up to 28-days. Scale bar = 2 mm.

The locations of 12 proteogenic amino acids (alanine, arginine, asparagine, aspartic acid, glutamine, glycine, histidine, leucine, proline, serine, threonine, and valine) were determined by ELDI-MSI. The non-proteogenic amino acids were pyroglutamic acid and GABA (Figure 10). Arginine, aspartic acid, and histidine were considerably more abundant in the shaded side of the leaf, although in the case of histidine this increase occurred after the half-leaf was shaded for 21 days. The spatial distribution of seven amino acids were unaffected by shading, but the relative abundance of four amino acids changed as time progressed. Specifically, leucine abundance increased at 28 days, whereas the abundance of alanine (decreased from day 14 to 28), glycine (peaked day 14 decreased from day 21 onwards), and proline (depleted by day 21) was reduced during this time period.

## Discussion

Several MSI methods have been utilized to obtain spatial distribution data of metabolites in plant samples. Each offers unique advantages and drawbacks (Lee et al., 2012; Boughton et al., 2016). The present work demonstrates the potential of applying ELDI for MSI. ELDI is analogous to LAESI, with the exception that ELDI uses ultraviolet radiation for ablation (e.g., 355 nm laser), whereas LAESI generally uses infrared radiation, for example a 2940 nm mid-IR laser (e.g., Nemes and Vertes, 2007). Although ELDI has previously been reported with fungi (i.e., *Ganoderma lucidum* and *Antrodia camphorate*) (Huang et al., 2012), there is only one other moderately extensive plant application study using ELDI, LAESI, or MALDESI (Etalo et al., 2015). The spatial resolution was limited to 500 μm in that work (Etalo et al., 2015). Many proof-of-concept experiments using LAESI-MSI on plants have been reported (Bartels and Svatoš, 2015).

Because plants accumulate large quantities of UV-absorbing molecules (e.g., flavonoids, chlorophylls, terpenes, phenolics) the plant leaf itself serves as a pseudo-matrix. Ablation of the plant pseudo-matrix enables ELDI-based observation of additional molecules that are not UV absorbers (e.g., sugars, amino acids, and organic acids). In both ELDI and LAESI methods, particles undergo post-ionization via interaction with ESI droplets, and the resulting ions are then extracted into the mass spectrometer. Although LDI without post-ionization via ESI can achieve much higher spatial resolution, issues associated with ionization transfer still need to be addressed (Hölscher et al., 2009).

**Figure 11:**
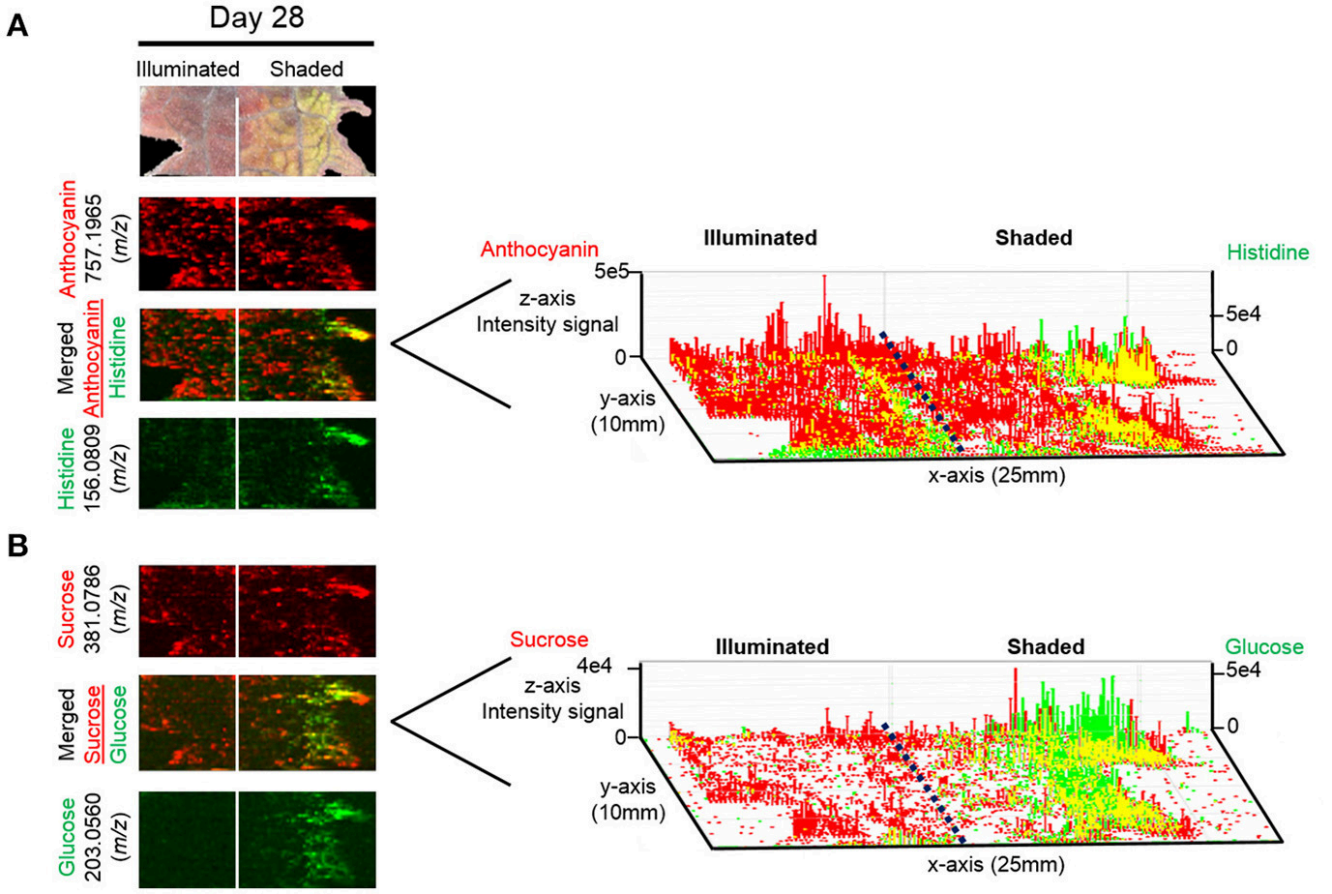
3D representation of the localization of UV-absorbing and non-UV absorbing metabolites. Distributions of **(A)** anthocyanin 757.1965 (*m/z*) (red) along with histidine 156.0809 (*m/z*) (green) and **(B)** sucrose 381.0786 (*m/z*) (red) along with glucose 203.0560 (*m*/z) (green) are shown. Scale bar = 2 mm. Right panel represents the 3D representations of the abundances of these metabolically related ions. The x- and y-axes represent spatial coordinates (mm), and the z-axis maps ion intensity.

One concern about our ELDI method is possible spatial heterogeneity of the UV-absorbing pseudo-matrix. Coupling electrospray and ablation with a UV laser appears to minimize these issues. Both UV and non-UV absorbing compounds can be measured at atmospheric pressure. For example, Figure 11 illustrates the localization of the UV-absorbing flavonoid cyanidin-coumaroylglucoside-glucoside (*m/z* 757.1965 [M^+^]) and the localization of the non-UV absorbing metabolites, histidine, sucrose, and glucose. Non-absorbing analytes can be observed readily in regions where the abundances of the pseudo-matrix flavonoids are relatively low (Figure 11). These comparisons indicate that ELDI-based MSI can be used to localize the distribution of non-UV absorbing metabolites despite large spatial changes in concentration of flavonoids or other possible pseudo-matrix compounds.

Coleus is convenient because its leaves are sectored visually. This reflects the spatial arrangement of the underlying flavonoids (Nguyen et al., 2008; Nguyen and Cin, 2009; Boldt, 2013). Moreover, in Coleus the sectoring of the adaxial surface is different from that of the abaxial surface. The adaxial surface is further modifiable by changes in the exposure to illumination (Nguyen and Cin, 2009; Logan et al., 2015). In addition, the depth of the leaf adaxial cells matches the penetration depth of the laser (~30 μm). Thus, the images presented herein reflect the abundance of metabolites in the epidermal cell layer of the leaf.

Correlated with the visible changes in the pigments of the leaf in response to shading, major metabolic changes in anthocyanins were visualized by ELDI-MS and quantitatively confirmed by LC-MS analysis of metabolite extracts. The cyanidin glycosides are the most abundant anthocyanins in the illuminated side of the leaves, which correlates with their photo-inhibitory potential (Steyn et al., 2002). Consistent with this photo-inhibitory attribute, the abundances of 17 of the 39 detected anthocyanins decreased upon shading of the leaf. Moreover, in response to the illumination status of the leaf, there is a coordinated change in the differential abundance of the structurally related anthocyanins, cyanidin-coumaroylglucoside-glucoside, cyanidin-malonyl glucoside, and the aglycone cyanidin. The ability to visualize this coordination at a spatial level provides additional insights to their potential metabolic interconnections. Namely, the enhanced accumulation of cyanidin-coumaroylglucoside-malonylglucoside, in response to illumination, may be associated with increased biosynthesis. The correlated changes in the levels of the potential precursors (i.e., cyanidin-malonyl glucoside) (Figure 7) support this hypothesis.

Multi-cellular photosynthetic plants are characterized by a series of source-sink tissues, that share the metabolic tasks of converting inorganic precursors, such as CO_2_, ammonia, phosphate etc., to organic constituents that are normally stored in sink tissues, such as seeds, tubers etc. (Turgeon, 1989; Basu et al., 1999; Roitsch, 1999; Paul and Foyer, 2001; Wardlaw, 2006; Lemoine et al., 2013; Osorio et al., 2014). Source-sink relationships can be genetically programmed and are further modified by environmental abiotic or biotic stimuli that are mediated by small molecules (e.g., sugars and amino acids) (Krapp and Stitt, 1995; Paul and Driscoll, 1997; Roitsch, 1999; McCormick et al., 2008; Lemoine et al., 2013). ELDI-MSI can image metabolic changes associated with the induction of an artificial new source-sink relationship between two halves of a leaf. The fully illuminated half of the leaf serves as the source tissue that fixes carbon, primarily in the form of sugars. These compounds are exported to the shaded half of the leaf that serves as the new sink tissue, whose strength increases with increasing time of shading (Figure 8). (Bagnall et al., 1988; Paul et al., 1992; Steyn et al., 2002; Islam et al., 2005).

ELDI revealed that the distributions of sugars (tetroses, pentoses, heptoses, and sucrose) were not significantly affected by the difference in the illumination between the two halves of the leaves. Only the distributions of glucose and a hexose phosphate (presumably glucose-6-phosphate) were affected; they increased on the shaded side (Figure 8). Therefore, the illuminated source-side of the leaf compensated for the reduced photosynthesis that was imposed by shading the other half of the leaf. This sugar-based interrelationship between source-sink tissues often manifests coordinated changes in amino acid metabolism, associated with the affiliation between carbon and nitrogen metabolism (Paul and Driscoll, 1997; McCormick et al., 2006). These changes in amino acid metabolism are usually associated with photosynthetic source tissues, where changes in RUBISCO levels (the major sink for amino acids) can drastically affect free amino acid pools (Paul and Driscoll, 1997; Nielsen et al., 2002; McCormick et al., 2008). Thus, in our studies we visualized increasing levels of Arg, Asp, His in the shaded side of the leaf (Figure 10), probably reflecting the turnover of RUBISCO as photosynthetic capacity was reduced in the dark, sink-side of the leaf.

## Conclusion

This study demonstrates the capabilities of ELDI-MSI for identification and spatial characterization of a wide variety of compounds in plant tissues with minimal sample preparation requirement. The ability to generate spatial distribution data that are consistent with biological explanations provides confidence in the validity of the observations. Future experiments include a) separation of isobaric ions by ion mobility, and b) implementation of procedures for quantification with spatial resolution by ELDI.

Compared to MALDI or other methods that require an added matrix, ELDI has the advantage of less sample preparation; however its sensitivity and spatial resolution are poorer. Much of the plant sample remains intact after ELDI analysis. Compared to LAESI with the commercial source, their spot size and spatial resolution are ~200 μm, while ELDI as shown here is ~125 μm. All plants that we have analyzed absorb at 355 nm well enough for laser ablation analysis. While the classes of compounds we report here were readily observed, other classes, such as lipids and peptides, were difficult to observe in our ELDI experiments.

## Author contributions

All authors listed have made a substantial, direct and intellectual contribution to the work, and approved it for publication.

### Conflict of interest statement

The authors declare that the research was conducted in the absence of any commercial or financial relationships that could be construed as a potential conflict of interest.
